# Fundamental care in the emergency room: insights from patients with life-threatening conditions in the emergency room

**DOI:** 10.1186/s12873-024-01133-4

**Published:** 2024-11-17

**Authors:** Veronica Pavedahl, Åsa Muntlin, Ulrica Von Thiele Schwarz, Martina Summer Meranius, Inger K. Holmström

**Affiliations:** 1 School of Health, Care and Social Welfare, Mälardalen University, Eskilstuna Västerås, Sweden Holmström, Eskilstuna Västerås, Åsa Sweden; 2 Department of Prehospital and Emergency Care and Area 3, Uppsala University Hospital, Uppsala, Sweden; 3 Department of Medical Sciences, Uppsala University, Uppsala, Sweden; 4 Department of Public Health and Caring Sciences, Uppsala University, Uppsala, Sweden; 5 Medical Management Centre, LIME, Karolinska Institutet, Stockholm, Sweden

**Keywords:** Interview study, Emergency care, Emergency department, Emergency room, Fundamentals of care, Person-centered care, Patient experiences

## Abstract

**Background:**

Persons who become life-threateningly ill or injured (due to for example trauma or cardiac arrest) are cared for in hospitals’ designated emergency rooms at the emergency department (ED). In these rooms, the life-threatening condition and biomedical focus may reinforce a culture that value the medical-technical care. Meeting patients fundamental care needs (integrating physical, psychosocial and relational care needs) in a person-centred way might hence be challenging in emergency rooms. Little is known about how person-centred fundamental care is experienced and valued by vulnerable and exposed patients in emergency rooms. This study aims to describe fundamental care needs experienced by patients with a life-threating condition in the emergency room.

**Methods:**

A descriptive deductive qualitative study with individual interviews were carried out with 15 patients who had been life-threateningly ill or injured and admitted in an emergency room, in Sweden. Data collection was conducted during 2022. Transcribed interviews were analyzed with deductive content analysis, based on the Fundamentals of Care framework.

**Results:**

Despite being life-threateningly ill or injured, patients were still able to describe their unique needs—which were not only related to biomedical care. A relationship was established between healthcare professionals and the patient in the initial stage, but not maintained during their stay at the emergency room. Patients felt their physical needs were met to a greater extent than psychosocial and relational needs, despite their prioritizing the latter. Patients preferred personalized care but described care as task oriented. The physical environment limited patients from having their fundamental care needs met, and they adopted to a “patient role” to avoid adding to staff stress. The emergency room situation evoked existential thoughts.

**Conclusions:**

This paper provides unique insights into patients’ experiences of being cared for in an emergency room. From the patient perspective, physical care was not enough. Relationship, timely and personalized information, and existential needs were identified as essential fundamental care needs, which were not, or only partly met. The finding highlights the need to embed and prioritize fundamental care in practice also for patients who are life-threateningly ill or injured, which in turn calls for focus on organizational prerequisites to enable person-centred fundamental care.

**Supplementary Information:**

The online version contains supplementary material available at 10.1186/s12873-024-01133-4.

## Background

Patients who suffer from life-threatening condition – experiencing illness or injury such as trauma, sepsis, respiratory problems, or cardiac arrest – are cared for at designated emergency rooms (called resuscitation rooms or trauma rooms in some countries) within the ED. A life-threatening condition is a condition that is considered so serious that the person’s life is threatened if he or she does not receive immediate care [1, 2]. In emergency rooms, the focus is often on biomedical and life-saving procedures, emphasizing medical status and technology [3]. The emergency room is designed and equipped to treat patients requiring immediate care, but it is not intended for longer time treatment. While advanced care is provided, patients who remain in a life-threatening condition after the initial assessment may be transferred to the operating room or intensive care unit for ongoing monitoring and specialized treatment [2]. Being admitted to the emergency room is an unplanned situation, occurring during a time of stress and uncertainty that leaves the person existentially and psychologically vulnerable, exposed and dependent. This vulnerability can result from factors such as organ failure, neurological impact, and difficulty in self-reporting, all of which necessitate medical and nursing care [4]. It is commonly observed that patients treated in emergency rooms are unable to articulate their needs due to severe pain, decreased level of consciousness or, hemodynamic instability [5].

The initial assessment and treatment in the emergency room is based on the Airway-Breathing-Circulation-Disability-Exposure concept (ABCDE) as well as Advanced Trauma Life Support concept (ATLS), providing a systematic, organized way of working to provide optimal care [6]. This quick and systematic approach has been described by patients as being strict and impersonal and, together with an acute health problem, the environment can feel stressful [7, 8]. Due to the demanding nature of the environment, coupled with a diverse array of patient needs to address, providing holistic care can pose challenges [8, 9]. One way of providing holistic care is using the Fundamentals of Care framework [10], addressing care in an integrated manner [11]. Fundamental care encompasses the essential support needed by everyone for survival, health, well-being, maintenance, protection, or a peaceful death, regardless of their clinical condition or the care setting [12]. Ensuring patients’ fundamental care needs are met in the emergency room is crucial for preventing both physical complications such as pressure injuries [13] and psychological complications, e.g., post-traumatic stress disorder [14]. Although Graham, Endacott [15] found that registered nurses (RNs) who are attentive to a patient’s needs are seen as providing a positive patient experience in EDs, a recent study [16] showed that RNs in the emergency room mainly met the patients’ physical needs, and communication with the patient gradually decreased during their stay in the emergency room. Thus, there seems to be a discrepancy between the fundamental care needs of the patients and the priorities of RN that requires further exploration.

## Theoretical framework

**Fig. 1:**
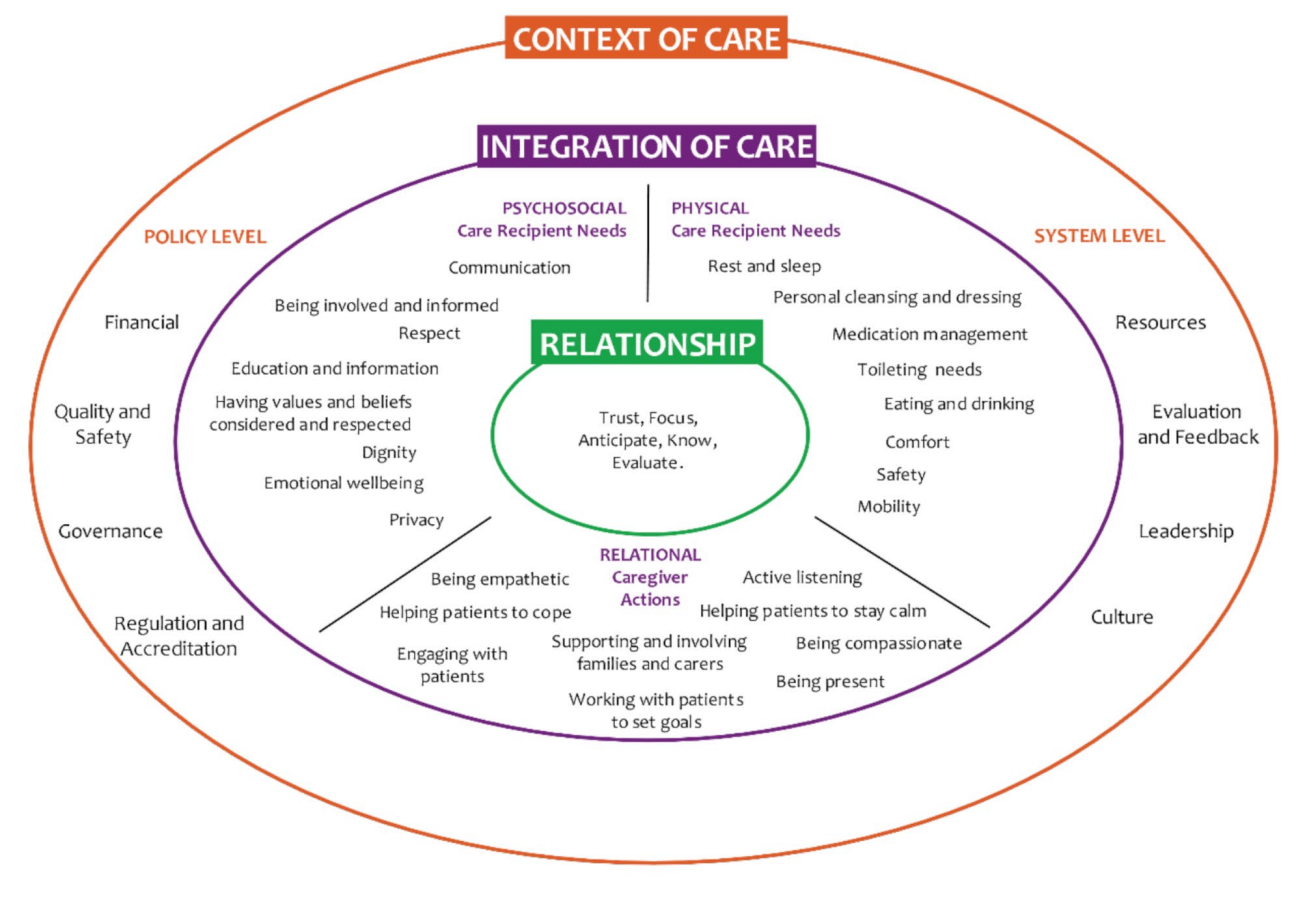
The Fundamentals of Care Framework [10] (Source: Feo et al. (2018), reprinted with permission).

The Fundamentals of Care framework [10] presented in detail in Fig. 1, consists of three interconnected dimensions: Relationship – establishing the caring relationship with the patient; Integration of Care – assessing and delivering physical, relational, and psychosocial fundamental care; and Context of Care – conditions in the form of factors at the system and policy levels for delivering these elements in a wider care context. By approaching the patient with a personalized understanding of their unique individual preferences, the nurse-patient relationship can be established. The RN needs to develop a relationship based on trust with the patient. This involves focusing on and anticipating the patient’s needs, comprehensively understanding their condition and continuously assessing and evaluating the quality of the relationship [17]. Based on the relationship, the RNs care provision towards meeting the patient’s physical and psychosocial fundamental care needs, mediated through relational actions, in an integrated way. The context, encompassing both system and policy level factors, could either facilitate or impede the provision of high-quality fundamental care [10]. Kitson [11] states that the Fundamentals of Care framework endorse the core values of person-centeredness and guides the practice of person-centered fundamental care. In other words, working based on the Fundamentals of Care framework enables the patient to receive person-centered care. Person-centered care is a key component of good quality care and a core competency for all healthcare personnel [18]. It is described as an ethical approach to care, taking a holistic view of the entire person [19]. The person-centered care approach places the person receiving care at the center and focuses on their needs, strengths, and weaknesses, and views the patient as an active part in the care and decision-making [19, 20].

Due to demographic shifts marked by an aging population, emergence of novel and demanding diseases like the COVID-19 pandemic, resource constraints, and a predominant biomedical orientation, RNs tend to deprioritize addressing the fundamental care needs of patients within the emergency room settings [21, 22]. Patients does not seem to receive the care they are entitled to, which can have consequences for their lives and on their continued care. Although patient’s experiences in general EDs have been studied, few studies to date have investigated patients’ experiences of being cared for in an emergency room context, when life-threateningly ill or injured. By studying patients’ experiences and mapping those experiences in the Fundamentals of Care framework, additional knowledge may be gained about fundamental care regarding life-threatening conditions within the emergency room context. Also, factors that may influence fundamental care in the emergency room context can be identified. The aim of this study was to describe fundamental care needs experienced by patients with a life-threating condition in the emergency room.

## Methods

### Design

This study utilized a descriptive deductive design with a qualitative approach [23], conducting individual interviews with patients to study their experiences. The Consolidated Criteria for Reporting Qualitative Research (COREQ) checklist was used for reporting on the process (supplementary file I). This study was part of a larger project aiming to investigate fundamental care in emergency rooms from different perspectives.

### Setting and sample

This study was conducted with patients who had been life-threateningly ill or injured in an emergency room situated in an ED at a university hospital in ‘REDACTED’. The ED receives about 54,000 visits annually, with over 3,000 of these involving emergency rooms admissions. The specific emergency room is located within the ED and has space for six patients. The healthcare professionals can screen off between the patient beds, using folding walls. Purposive sampling was used to facilitate variability with regards to sex, age, and reasons for seeking care. Potential participants were identified from the ED’s patient tracking system. Potential participants were informed about the study, and invited to participate by letter. One week after the letter was sent out, they received a follow-up phone call and asked to participate. The invitation letter was distributed to 54 individuals during a designated period. The inclusion criteria were being 18 years of age or older, and the ability to understand and speak the Swedish language. Exclusion criteria were persons still admitted to in-hospital care, expected persistent cognitive failure, suicide attempt, and intoxication. These exclusion criteria were applied for ethical reasons. Patients were contacted for recruitment within a period of 14–30 days of being discharged from in-hospital visit. The participants consisted of 15 persons, six women and nine men. They ranged in age from 32 to 84 years (mean: 65 years). Commonly alerted from the ambulance triage, participants arrived in the emergency room for different reasons, (e.g., heart failure (*n* = 3), pneumothorax (*n* = 1), hypertension (*n* = 1), hypoglycemia (*n* = 1), syncope (*n* = 1), chest pain (*n* = 2), shortness of breath (*n* = 2), sepsis (*n* = 1), fall (*n* = 1), epileptic seizures (*n* = 2)). After stabilization in the emergency room, patients were most commonly transferred for further care (e.g. intensive care, operation theater, general ED). The participants in this study spent 60 min or more in the emergency room.

### Data collection

A semi-structured interview guide based on the Fundamentals of Care framework was used, developed for this study (supplementary file II). The initial question investigated the patients’ experiences of how their fundamental care was executed in the emergency room. Examples of questions include: *“Can you describe your experience of being cared for in the emergency room?”; “Can you describe the encounter/relationship with the RN?”; “Can you describe the needs you had*,* and do you feel that they were met?”* and *“Can you describe what worked well/can be developed in the emergency room?”*. The interview guide was pre-tested with a single participant, who was subsequently excluded from the final study sample. Following this pilot interview, only minor revisions of the interview guide were done. Patients were encouraged to openly share their experiences regarding treatment and care in the emergency room. The interviewer asked follow-up questions and sought clarification when needed. We asked specifically about the RNs caring; however, the participants answered about the care provided by healthcare providers in general. All the interviews were conducted by the first author (‘REDACTED’). (‘REDACTED’) was not familiar with the ED nor the participants, however an RN with substantial experience in managing patients with life-threatening conditions. Due to the COVID-19 pandemic, the interviews took place by telephone and lasted from 13 to 40 min (mean: 30 min). The interviews were audiotaped and transcribed verbatim by the first author. Data collection was conducted from April-May 2022.

### Data analysis

Data were analyzed using qualitative content analysis according to Elo and Kyngäs [24], who provide a description of deductive content analysis, in phases of preparation, organizing and reporting. In the first phase according to Elo and Kyngäs [24], the authors read and re-read the transcripts to familiarize themselves with the dataset. The transcripts were examined for content and meaning units belong to the three dimensions *(relationship*,* integration of care and context of care)* of the Fundamentals of Care framework, which were then extracted and coded under the corresponding dimensions. A categorization matrix (presented in detail in Table 1) was constructed, based on the Fundamentals of Care framework [12], and used as a lens for the analysis process. The findings were then organized into categories derived from the deductive analysis, considering the research objectives. The analysis involved a constant moving back and forth within the entire data set. See Table 1 ‘Categorization matrix of the experiences of patients with life-threatening conditions related to fundamental care in the emergency room, based on the Fundamentals of Care Framework’ for the steps of the data analysis.

**Table 1:** ‘Categorization matrix of the experiences of patients with life-threatening conditions related to fundamental care in the emergency room, based on the Fundamentals of Care Framework’

Dimensions from the Fundamentals of Care Framework	Categories	Codes
Relationship	I want to know who you are See me, I am not an object	The staff need to introduce themselves by name and role Want to know who in the staff I’m talking to Need to gain trust in the staff Trust in staff who seemed competent Lonely despite presence of staff Treated kindly, with respect Want the staff to show focus Not seen as a person, being objectified
Integration of care	Relational caregiver actions: Maintain the relationship with me throughout my stay in the emergency room Psychosocial care recipient needs: I feel a lack of privacy and integrity Psychosocial care recipient needs: I am in need of continuous, timely and personalized communication and information Physical care recipient needs: I am in discomfort, but I cannot move or see you Physical care recipient needs: I am in need of personalized and timely pain management	Engagement decreases The staff focuses on the medical aspects Physical needs met, not psychosocial Non-privacy Mobility and field of vision is reduced Lack of personalized information and communication Not being able to bring relatives Not being able to move Being placed facing a wall Low degree of involvement Non-optimal analgesics Lack of personalized pain management
Context of care	I adapt to a patient role by taking the healthcare providers’ situation in consideration I sense a proximity to strangers I am not familiar with the emergency room organization	High presence of staff Don’t want to disturb the staff Don’t want to add to staff stress Hear other people die The emergency room evokes existential thoughts The staff have your life in their hands After being disconnected from monitoring equipment no one cares about you Chaotic, stressful environment Bizarre place

### Ethical considerations

The project was approved by the Swedish Ethical Review Authority (Dnr. 2019 − 00506) and followed the guidelines of the Helsinki Declaration [25]. Participants received verbal and written information about the study and provided informed consent before participating in the study. They were informed that their participation was voluntary and that they could withdraw from the study at any time without having to provide a reason. Data were processed and stored to maintain confidentiality. Personal information, including informed consent records, was safeguarded in a locked safe. Electronic data were stored on a password-protected computer. All collected data adhered to the General Data Protection Regulation [GDPR] [26] following rules and regulations of the University in question.

### Rigor

COREQ were applied for the reporting of this study [27]. During the coding sessions, all team members were involved in continuous discussion about the data throughout the analysis process adding to credibility. Dependability was ensured through an audit trail, and confirmability was strengthened by using quotes from the interviews. It might be possible to transfer the findings to other patients’ experiences of being cared for in the emergency room. Before data collection, the first author’s prior understanding as an RN was thoroughly discussed and considered by the other authors. The co-authors of this study are experienced in research and clinical work involving nursing care, emergency care, and psychology and provide a broader health service perspective.

## Results

The findings describe fundamental care needs in the emergency room, based on experiences of patients with life-threatening conditions. The findings are presented according to the three dimensions (relationship, integration of care, and context of care) from the Fundamentals of Care framework [10] in 10 categories. The themes are presented in the voice of a patient to emphasize a person-centered approach.

### Relationship: built on trust, focus and knowledge

The dimension “Relationship” includes the following categories: *“I want to know who you are”* and *“See me*,* I am not an object”.* When life was at stake, feeling of trust was crucial. Even though assessment and intervention had to be done quickly and systematically by healthcare providers, patients expected to be treated kindly and with respect. Healthcare providers who had knowledge about the patient and provided thorough and comprehensive care, built trust in the patient. Trust promoted the relationship and generated a sense of security and “being seen” in a vulnerable situation.

#### I want to know who you are

Patients wanted to know who cared for them. They found it frustrating when healthcare providers only introduced themselves by name but not by role. Knowing the roles of the healthcare providers provided a sense of trust, safety, and gave them a choice of who to share information with.

*“People came to talk to me and all that*,* and maybe they introduced themselves*,* but you never knew if it was a janitor*,* a physician or a nurse or what kind of person it was. Maybe I would feel calmer if I knew who I was talking to”* (participant no. 1).

#### See me, I am not an object

One important feature of the relationship in the emergency room was when the healthcare providers focused on the patient as a unique person. In a life-threatening situation, being able to explain one’s situation, express personal needs and have concerns taken seriously led patients to feel seen and relaxed in the hands of the healthcare providers. On the other hand, being referred to by the name of one’s medical condition was perceived as dehumanizing and objectifying. As one participant stated:

*“The personal contact will be what it will be*,* you’ll only be there for an hour or so but… To a certain extent*,* I felt that they (the healthcare providers) talked about me as ‘yes*,* she’s the one with the heart’…So*,* I understand but at the same time*,* I missed the personal aspect*,* even if it was a short encounter”* (participant no. 5).

Healthcare providers who gave the appearance of focusing on devices rather than the patient created a feeling of not being interesting, taken seriously, or seen as a person. The focus was on medical data and procedures, not the patient, as illustrated in the following quote:

*“They put a needle or whatever it’s called in my arm*,* without telling me. They did not tell me what they were doing*,* it was as if they were*,* yes*,* they wanted to concentrate on work and not me. How I felt in that situation did not come in first hand”* (participant no. 8).

### Integration of care: fluctuating integration of care needs

The dimension “Integration of care” includes the following categories: *“Relational caregiver actions: Maintain the relationship with me throughout my stay in the emergency room”*,* “Psychosocial care recipient needs: I feel a lack of privacy and integrity”*,* “Psychosocial care recipient needs: I am in need of continuous*,* timely and personalized communication and information”*,* “Physical care recipient needs: I am in discomfort*,* but I cannot move or see you”* and “*Physical care recipient needs: I’m in need of personalized and timely pain management*”. Experiences of physical, relational, and psychosocial fundamental care needs were vividly described. Patients wanted their care to be delivered in a holistic manner and not only be medically treated.

#### Relational caregiver actions: maintain the relationship with me throughout my stay in the emergency room

In an unforeseen and distressing situation, healthcare providers who were present, and assessed and measured the patient’s needs and symptoms without a stressful approach helped the patient to stay calm. Commitment throughout the entire care process was highly valued, however, attention gradually decreased as the patient’s condition improved. After the initial assessment the number of healthcare providers reduced. They did not engage and were no longer present by the patient’s side to the same extent, which in turn created feelings of abandonment and being neglected.

*“It was weird in a sense*,* it was very intense in the beginning*,* and then I probably wasn’t the sickest. However*,* there was still a lot of healthcare professionals in the room*,* but they were not with me. No one were with me*,* I was alone and then*,* then the thoughts came…I realized that I had been hours from death (silence). I didn’t really understand*,* it felt a bit overwhelming*,* shocking*,* that I was close to die* (participant no. 3).

#### Psychosocial care recipient needs: I feel a lack of privacy and integrity

The experience of fundamental care needs in the emergency room was associated with a lack of privacy and integrity. Although patients were curtained off, the curtains did not offer sufficient protection, as they could hear everything that the staff and other patients said. To lie there and listen without wanting to hear was uncomfortable as it felt like eavesdropping and intruding in someone else’s life, as illustrated in the following quote:

*“I was lying by a door*,* but they put up a screen facing the corridor and then there were screens between the person lying next to me. But it’s still just a screen*,* so you hear everything that the others say*,* and you hear the healthcare providers when they speculate among themselves and about other patients. You hear everything that happens around you*,* even if you do not want to. Like later in the evening I heard it was a guy who was quite young who came in with a heart defect*,* and yes*,* he was completely devastated…I wanted to*,* like shut my ears”* (participant no. 4).

What affected the patients’ integrity was often connected to actions of healthcare providers, for example, if the healthcare providers did not close the curtain behind them or spoke quietly so that others would not hear. When healthcare providers failed to provide privacy, it led to feelings of discomfort, as dignity had been compromised. This is illustrated in the following quote:

*“I got a blanket in the end*,* but in the beginning*,* I was in my pants and shirtless. When exactly the blanket came*,* I can’t remember. Ehh*,* it wasn’t that you were lying completely…visible*,* but it was more the feeling that there were others lying in there and it was open to the corridor with people walking by. Also*,* I had to pee in a bottle and then I had to stand up*,* probably visible to others. It felt a bit undignified”* (participant no. 9).

#### ***Psychosocial care recipient needs: I am in need of continuous***,*** timely and personalized communication and information***

Communication was experienced as being one-way; the healthcare providers were the ones who asked questions and the patient answered. Regarding communication, there was a need for continuity among the healthcare providers. As the situation itself was overwhelming, repetition of symptoms to different healthcare providers became cognitively challenging. Having to explain one’s situation repeatedly to different the healthcare providers was tiring and created a concern about leaving out of something important, as described in the following quote:

*“Many different healthcare providers came and asked the same thing. Repeating the same information over and over again was a bit frustrating…I became stressed and worried that I would forget to tell things*,* thing that was important for my condition.”*(participant no. 5).

The need for information was frequently brought up. Although information was given, but there was a need expressed for clear, repeated, personalized information, not a standard “on-size-fits-all” explanation. Information given exactly when a procedure was being done gave the patient no opportunity to process the information nor prepare for what was about to happen. Receiving information when not receptive or in a vulnerable position led to increased anxiety and misunderstanding. When life-threateningly ill, there was sometimes a need to leave decisions to the healthcare providers. It seems that this was a preference of (at least some) patients (i.e., to have the healthcare providers make decisions in times of crisis). As one participant stated:

*“They were talking to me all the time and asking me things*,* but it was difficult to answer all the questions because I was so out of breath. I was a bit dizzy at times. I just wanted to leave all decisions to them*,* I can say I didn´t care about…I didn’t care about anything other than getting well again”* (participant no. 12).

#### Physical care recipient needs: I am in discomfort, but I cannot move or see you

Because of the monitoring equipment connected to them, patients were commonly placed in a supine position which dramatically reduced mobility, field of vision, and comfort. Staring at the ceiling without being able to turn and see the healthcare providers or what was going on in the room was anxiety provoking and discomforting. One patient said:

*“All the healthcare providers were behind me the whole time*,* so I couldn’t see them. I became a bit stressed by staring at the ceiling*,* not knowing what was going on. They (the healthcare providers) had to come forward and show themselves when they wanted to see me…It felt inconvenient not being able to see the healthcare professionals when you wanted to*,* even though you could hear them“* (participant no. 9).

#### Physical care recipient needs: I am in need of personalized and timely pain management

Suffering from pain and the need for analgesic was associated with a need not being met. To get analgesics, patients described that they had to exaggerate their pain. The effect of any analgesic given was often not evaluated, therefore patients could continue to be in pain without being offered further analgesics. Not receiving optimal pain relief made the situation seem unbearable and of the patient feel powerlessness, as illustrated in the following quote:

*“The pain was the worst. Not getting any help to get rid of it. It was…I wanted to jump out*,* you know. It felt unbearable. I wondered if there was…*,* could there have been other analgesics they could have tried because the pain was so intense. But I was…I didn’t ask and they didn’t offer anything else so probably there wasn’t”* (participant no. 10).

### Context of care: a stressful and surreal context

The dimension “context of care” includes the following categories: “*Adapting to the patient role by taking the healthcare providers’ situation into consideration*”, “*I sense a proximity to strangers*” and “*I’m not familiar with the emergency room organization*”. Being a patient in the emergency room environment was characterized by various ambiguities, as the emergency room environment was described as terrifying, bizarre, and noisy —yet high-tech.

#### I adapt to a patient role by taking the healthcare providers’ situation into consideration

Due to the environmental challenges of the emergency room, patients were prevented from calling attention to and expressing their needs. Patients took the healthcare providers’ situation into consideration by not expressing certain needs if they noticed that the healthcare providers were stressed. For example, the patients stated that they did not want to disturb unnecessarily or add to healthcare providers stress and, therefore, did not ask, for example, for a blanket. Being offered a bottle to urinate in, instead of being helped to the toilet, made the patient prefer to wait. This further contributed to their vulnerable situation. Adapting to the patient role was described in the following quote:

*“I understood that they were busy with important things*,* so I didn’t mention how thirsty I was. I really wanted something to drink*,* but I did not ask because I was afraid I might have to pee. I was tangled in the monitoring cables and did not know if I would have time to get help to the toilet in time. Even though I was so thirsty*,* I did not want to risk wetting the bed”* (participant no. 8).

#### I sense a proximity to strangers

Hearing other patients fight for their life and die in the room was traumatic, while at the same time a feeling of gratitude was described that one’s own circumstances were better. Patients compared their situation to other patients in the room. Shocking events involving fellow patients made a strong impression; the emergency room, and the situation they were in, evoked existential thoughts, such as thoughts about one’s own death:

*“The person probably had five or six members of the healthcare team around him*,* pumping life into this person*,* they ran with oxygen*,* they did everything… Then you lay there and listen*,* ‘we’ll try again’*,* ‘no’*,* ‘we’ll try again’*,* ‘no*,* now we’ll probably have to give up’. And then*,* yes*,* then you heard that it was over. It was like being in a movie; was I going to die too? I’m grateful that I got out of there”* (participant no. 1).

#### I am not familiar with the emergency room organization

Despite the constant presence of enumerable resources in the form of healthcare providers, there were fluctuations in activity in the emergency room. Patients experienced a constant movement of healthcare providers in the emergency room as such, however, they did not always find a healthcare provider at their own bedside. After the initial care, or when their condition had improved, patients came to realize that they were no longer the most interesting case and often had to wait to be addressed. It was unclear to the patient who was responsible for their care, which was perceived as a lack of organization and leadership. One patient described:

*“So*,* in the beginning there were people everywhere doing different things. Then*,* as quickly as they came*,* they disappeared without telling. And suddenly everything felt so provisional*,* with no sense of organization behind it. No one asked me anything anymore; I just lay there*,* maybe not forgotten but passive. It felt like I was in a field hospital*,* it was chaos but still a bit order in some weird way”* (participant no. 13).

## Discussion

The aim of the present study was to understand the fundamental care needs in the emergency room, based on experiences of patients with life-threatening conditions. The perspective of this group of patients is rare in the literature, and this study shows that despite being life-threateningly ill, patients were still able to describe their unique needs—and these were not only related to medical care. This study highlights that the patients’ experience of fundamental care in the emergency room is multifaceted, and they expressed several unmet fundamental care needs during a time of vulnerability.

Within the dimension relationship, patients highlighted being seen and treated like a person with individual needs, which is a prerequisite for person-centered care [19]. Health care providers who had knowledge about the patient and provided thorough and comprehensive care seemed to create feelings of trust, which made the patient feel respected. However, when the RNs adopted a task-oriented approach, the patient felt objectified, leaving their personal needs unmet. In line with recent research [7], patients valued the opportunity to be involved in their care. Even in a life-threatening situation a sense of participation could be enhanced. For care to be person-centered within the emergency room care context, the healthcare providers have a responsibility to treat patients as persons, and meet their physical, psychosocial, and relational needs in a holistic manner, that is, not focus only on medical aspects [28, 29]. This might be challenging given the context but should be a goal to strive for.

Patient and RNs perspectives regarding the prioritization of physical versus psychosocial and relational needs may exhibit disparities. In the current study, patients appeared to emphasize psychosocial and relational needs over physical care requirements. This emphasis may arise because physical care is often perceived as routine and expected, leading patients to assume that they will automatically receive optimal medical attention. However, the need for patients to have their emotional needs addressed was emphasized by the interviewed patients. It should however be noted that there is a difference in how patients experience care during their stay and how they reflect on the received care afterwards. The wish patients expressed to prioritize psychosocial care over physical care should be seen in light of their reflections on the care at the emergency room. In contrast, from the RN’s perspective, patients’ physical needs are prioritized in the emergency room, with less attention given to the relational and psychosocial needs, and patients’ existential issues were not identified and acknowledged [16]. A recent study showed that even though it is common to have feelings of stress, anxiety and fear when entering an ED, patients do not share their emotional concerns with the RN [9], which is in line with the present findings. While emergency room RNs naturally prioritize life-saving interventions, it is essential to deliver care in a holistic manner. RNs have a crucial role in recognizing and addressing the comprehensive needs of patients and in planning their care. This study demonstrates that even patients in life-threatening conditions have needs that extend beyond the physical ones, as the severe nature of their situations also brings about existential thoughts and feelings. Thus, having one’s medical needs met could be described as being necessary but not sufficient for patients satisfaction and high-quality care delivery. The lack of emotional support could cause extra vulnerability and suffering in already stressed patients [30].

For example, hearing or witnessing traumatic events involving fellow patients in the emergency room needs to be addressed with adequate emotional support.

We found that the patients experienced most attention in the initial phase of the care process, however, after the initial care delays in receiving further care could occur. This is in line with findings showing that RNs initially focused on the patient, specifically their physical needs, however, this decreased in the course of care [31]. Establishing a relationship is central to the Fundamentals of Care framework [10], and from a person-centered care perspective partnership is fundamental [19]. Nevertheless, the absence of essential conditions conductive to fostering patient relationships, RNs may default to standardized procedures, thereby hindering the delivery of personalized care required by and for patients. Therefore, extra focus is needed on how fundamental care can be promoted in a person-centered way after the initial assessment in the emergency room. This calls for both nursing leadership and guidelines. As patients in the emergency room are in a vulnerable situation, nursing care should be better clarified and receive more emphasis. Notably, the initial care for patients in life-threatening conditions in the emergency room is structured and systematic through the ABCDE concept and ATLS [6] but the results from this study reveals a lack of structure and clarity regarding fundamental care. In the emergency room context, there seems to be no consensus on how (or even on the fact that) patients’ fundamental care needs should be addressed. Maybe F, for fundamental care, should be added to ABCDE to show the importance of integration of fundamental care. However, that might also reinforce beliefs that F happens after ABCDE, whereas the point is that F highlights RNs specific responsibilities in the team throughout assessment and treatment.

Patients in our study highlighted that the lack of timely information and communication in a personal way negatively influenced the experience of having their fundamental care needs met in a person-centered way. The findings seem to correspond quite well with the findings from a general ED, of how communication is imperative for meeting patients’ fundamental care needs [32]. International research [15] has consistently highlighted the importance of providing patients with information, such as on waiting times, their condition, and the next step in the care, in reducing frustration and anxiety. Furthermore, our findings show that receiving information when patients were not in a position to be receptive or in a vulnerable situation, having information provided about others or having information about themselves by being referred to as a medical condition might led to increased anxiety and misunderstanding. This is confirmed in Egerod, Bergbom [33] showing that access to high-quality, personalized information given repeatedly is associated with less anxiety due to an increased feeling of control. Based on a person-centered approach [19], the patient should receive targeted information adapted to their personal needs to be able to achieve an adequate understanding of their situation and improve recovery outcomes. In the emergency room context, it is not always about having more information, but rather appropriate and timely information.

Being in a life-threatening situation, patients may lose control over their own situation, and become totally dependent on the health care professionals, whereupon the RN has a decisive role in identifying the patients’ needs and making a plan for their care. We found that the participants adapted to the patient role in the emergency room, which affected their ability to express their fundamental care needs. Patients commented on poor pain management and the lack of analgesics but instead of asking for an analgesic, they displayed a tendency towards tolerating pain. Patients hence chose not to ask about their unmet fundamental care needs as they did not want to disturb the health care professionals when they saw that the they were stressed. This is in line with Bull, Latimer [8] who showed that there is a power imbalance between the patient and the health care professionals in general EDs. Some patients felt that they needed to prove themselves worthy of being in the ED by presenting them as the best possible patient. According to the Fundamentals of Care framework, the context of care should be seen in terms of prerequisites and resources needed to ensure safe and high-quality fundamental care [10]. To provide the best possible conditions for promoting patients’ health, the staff work environment, health, and commitment are important [34]. A crucial determinant is the organizational culture of the care unit, characterized by prevailing values. Embracing a task- and time-oriented approach could perpetuate a culture that prioritizes medical and technical aspects over nursing, potentially leading to a heightened likelihood of neglecting nursing care for patients with life-threatening conditions. Recognizing a person in need of holistic care as opposed to viewing the patient as a name or condition to be removed from a task list promptly represents distinct approaches to nursing care.

Our results showed that patients who have been life-threateningly ill and treated in an emergency room, experienced, and talked about existential needs, which is not a dimension or element in the Fundamentals of Care framework. The new knowledge generated in our study requires further research regarding existential needs in patients’ suffering from a life-threatening condition and how it can be incorporated in the framework.

### Study strengths and limitations

One researcher conducted all the interviews, but to avoid bias all the research team members participated in the analysis process. Two of the authors have experience of caring for patients with life-threatening conditions, and this preunderstanding might have affected the analysis. However, the interviewer was not familiar to the hospital and had no relationship with the RNs who had taken care of the patients, or the patients interviewed. One limitation is that due to the COVID-19 pandemic, the interviews were conducted over the phone. This limited the opportunity of examining non-verbal communication. As the study was conducted during the pandemic, this might have influenced the participants’ experiences of care, although it was rarely mentioned during the interviews. This study was ethically challenging in several ways as it enrolled vulnerable participants, and hence, patients with cognitive failure, or admitted for suicide attempts or intoxication, were excluded. There was a period of 14–30 days between hospital discharge and participant recruitment, potentially introducing recall bias. However, this period was necessary because interviewing participants still admitted to hospital would have been impractical and unethical as it might have interrupted their care. It should also be mentioned that situations evoking strong emotions often are vividly remembered also after a long period of time. As the interviews were held at a large university hospital transferability to other, similar emergency rooms are possible.

## Conclusions

This study adds new insights into the gaps in literature concerning how patients with life-threatening conditions reflect on their fundamental care needs, in the emergency room. From the patients’ perspective, when being life-threateningly ill establishment of a relationship based on trust is crucial. However, there is a need for improvement in maintaining the relationship during the care period, as a decline in attention and care, resulted in feelings of neglect and uncertainty. Patients preferred personalized care, but described nursing care as being task-oriented. The environment of the emergency room further impeded patients’ ability to communicate their needs effectively as they were shielded only by folding walls from other staff and patients and were laying on trolleys looking into the ceiling. The lack of organizational structure left patients feeling overlooked, underscoring the need for improvements. The finding highlights the need to embed and prioritize fundamental care in practice also for patients with life-threatening conditions, which in turn calls for focus on organizational prerequisites to enable person-centred fundamental care. The knowledge can be used in emergency care practice to empower and facilitate person-centred fundamental care. The results can also be used to design education and teaching of RNs.

## Electronic supplementary material

Below is the link to the electronic supplementary material.

Supplementary Material 1

Supplementary Material 2

## Data Availability

Data generated during and/or analyzed during the study are not publicly available due to ethical restrictions and privacy.

## Abbreviations

ED: Emergency Department
RN: Registered Nurse
ABCDE: Airway Breathing Circulation Disability Exposure
ATLS: Advanced Trauma Life Support
