# Supplementary material for: Fundamental care in the emergency room: insights from patients with life-threatening conditions in the emergency room
Source: BMC Emerg Med. 2024 Nov 17;24:217. doi: 10.1186/s12873-024-01133-4 (PMC11571529; doi:10.1186/s12873-024-01133-4)
Supplement: Supplementary file 2 — Supplementary Material 2 [file 12873_2024_1133_MOESM2_ESM.pdf]

Questions

- Do you consent to participate in the study?
- How old are you?

- If you think back – can you describe the situation that led you to end up in the emergency room?
    - Tell me more about how it went when you arrived at the emergency department/room – What happened? What was done?
- Can you describe your experience of being cared for in the emergency room, of the initial care you received?
- How did you experience the interaction/relationship with the nurse? (or the staff)
- If you think back again – what needs would you say you had?
- How did you feel your needs were met?
    - *Physical:*
    - Did you get to go to the toilet? How did it feel (if the person did not receive help with toilet visits)?
    - Did you receive any food/IV fluids?
    - How was it with pain relief?
    - Could you move as you wanted? Did you get help to lie comfortably?
    - *Psychosocial:*
    - Did you feel that the staff communicated with you?
    - Did you feel that you received information? The information you needed?
    - Did you feel involved in your care? Can you describe how?
    - How was it with privacy? Were you covered with clothes/blankets?
    - *Relational needs:*
    - When you talked to the nurses, did you feel that they listened?
    - When you talked to the nurses, did you feel that they were present?
    - Did you have any relatives with you? Do you have any perception of whether they were supported and involved?
- How did you find the environment in the emergency room (noise/light/hard beds etc.)?
- Was there anything you thought worked well?
- Now that you might have some perspective on the event – do you see anything that can be improved? What could have been done differently for you in the situation you were in?

Follow up questions

What does it mean when you say…?

How did it feel to/when?

Can you explain/tell more? What happened? What did you do?

Can you provide a specific example?
